# Death-Bed Repentance

**Published:** 1860-03

**Authors:** 


### DEATH-BED REPENTANCE.

An old negro was on his dying-bed. Some one had done him a great injury, the forgiveness of which his faithful minister had labored hard to induce him to profess. At length, when just on the verge of the border-land, a strong last appeal was made:

"Tom, won't you forgive him?"

"Well, Massa, if I'm going to die, I suppose I must; but if I ever do get well, I'll give him another dig."

Our own impression, from long and special observation, is, that death-bed repentances have no reliable value; it is the repentance of desperation; there is no alternative, but that of the preached perdition! the straw is eagerly clutched at spasmodically, and not with a clear, discriminating, and intelligent faith. The whole Bible gives but one saving case; one, that none might despair; only one, that none might presume!

Of all living men, the physician feels most deeply that a sick-bed is the unfittest of all places for that mental composure which must be essential to a proper attention to the "great concern."

In this light, the parading of the professed contrition of criminals through the newspapers is most injudicious; it is an unmixed evil. Its tendency on the minds of the living, the desperate especially, is pernicious. The soliloquy runs thus: "I knew him well. He was a scoundrel of the deepest dye, yet he died happy, and I can do the same thing—live a rascal and die a saint." Thus the fear of death and retribution is blunted, and the way paved for a greater abandonment to all wrong-doing. Hence the clergyman who steps in, and allows himself to be made a tool of in this regard, desecrates his holy office, and must be pitied for his ignorance or despised for his presumptuous impertinence.

A most remarkable case of this kind has occurred within a few days. Two clergymen, whose names were unknown to fame before, and we do not propose by mentioning them, to illustrate them into a greater obscurity, published in the daily papers, under their own signature, that the man just hanged was in their opinion innocent of the crime charged against him, and that they believed he died a Christian. Such a declaration was equivalent to bringing the law into contempt, a thing which a good citizen will never do. It was calculated to foster a spirit of hatred on the part of the friends of the deceased, and very many others, against the sheriff, jury, judges, the Governor of the State, and against law itself, which is the shield of all good men, and, as Paul says, "The ordinance of God."

This man was hanged for murdering his wife by administering poison, indicating the utmost deliberation—giving her poison while nursing her in her sickness! she, in her weakness, and all confiding, receiving it in love, as a means of cure. It is difficult to conceive of a more unpardonable crime. The poison was found in her stomach after death. The jury condemned him; the judge acquiesced in the wisdom of their decision. The case was tried a second, if not a third time, with the same result. It was then taken from court to court, with the same unvarying verdict. The Governor of the State was appealed to in the last extremity, known of all, especially in the city of New-York, to be humane and generous beyond most men of his time; but he is also known to be judicious and inflexibly just. He declined to interfere with the course of the law, because he saw not a single point which could justify him in the exercise of his authority—not coming to this conclusion until he had carefully examined the whole case, with the aid of his official counsellor. And yet, here are two men, with a presumption literally unparalleled, who come forward and give the public, who had never heard of them before, their opinion, that this man was judicially murdered, because—he said he was innocent! when up to within forty-eight hours of his execution he had carried a revolver, either to shoot himself or the warden—fiercely denying, with pretended indignation, that he had a deadly weapon at all, which, however, he confessed he did have, when the officer took it out of his pocket! "He said he was innocent!" as if a man who would poison his wife, and carry a revolver for weeks, seeking an opportunity to use it on the warden of the prison, when by it a chance of escape occurred, as if such a man's word was to be believed! If clergymen begin to aid in bringing the law of the land into contempt in this and other ways, the sooner all good people abandon it, the better. For if the law does not reign supreme, crime, debauchery, unthrift, destitution, disease, and death will. If the watchmen sleep, who shall guard the shepherds?

